# PI3Kgamma Inhibitor Attenuates Immunosuppressive Effect of Poly(l‐Glutamic Acid)‐Combretastatin A4 Conjugate in Metastatic Breast Cancer

**DOI:** 10.1002/advs.201900327

**Published:** 2019-04-18

**Authors:** Hanjiao Qin, Haiyang Yu, Jiyao Sheng, Dawei Zhang, Na Shen, Linlin Liu, Zhaohui Tang, Xuesi Chen

**Affiliations:** ^1^ Department of Radiotherapy the Second Hospital of Jilin University Changchun 130041 P. R. China; ^2^ Key Laboratory of Polymer Ecomaterials Changchun Institute of Applied Chemistry Chinese Academy of Sciences Changchun 130022 P. R. China; ^3^ Jilin Biomedical Polymers Engineering Laboratory Changchun 130022 P. R. China; ^4^ Department of Hepatobiliary and Pancreatic Surgery the Second Hospital of Jilin University Changchun 130041 P. R. China

**Keywords:** combretastatin A4, immunotherapy, nanomedicine, tumor‐associated macrophages, vascular disrupting agents

## Abstract

Vascular disrupting agents (VDAs) have great potential for cancer treatment. Poly(l‐glutamic acid)‐combretastatin A4 conjugate (PLG‐CA4) is a novel class of VDAs. Though it has notable antitumor activity, it can induce host immune responses that promote tumor growth. Here, PLG‐CA4 induces the polarization of tumor‐associated macrophages (TAMs) toward the M2‐like phenotype in 4T1 metastatic breast cancer (Control 30% vs PLG‐CA4 53%; *p* < 0.05). Compared to the monotherapy of PLG‐CA4, inhibition of phosphoinositide 3‐kinase gamma (PI3Kγ) attenuates the immunosuppressive effect of PLG‐CA4 treatment by decreasing the number of M2‐like TAMs (2.0 × 10^4^ to 1.5 × 10^4^ per tumor) and potential enhancement of cytotoxic T lymphocyte (3.0 × 10^4^ to 5.7 × 10^4^ per tumor). Importantly, PI3Kγ inhibitor synergizing with PLG‐CA4 significantly extends the mean survival time from 52 days in monotherapy‐treated mice to 61.8 days. Additionally, the combination of PLG‐CA4 and PI3Kγ inhibitor improves the tumor therapeutic effect of NLG919, an inhibitor of immune checkpoint indoleamine 2,3‐dioxygenase (IDO). As far as it is known, this is the first demonstrated study that VDAs induce the reshaping of macrophages to the M2‐like phenotype. The findings also indicate a potential therapeutic strategy of the combination VDAs with an accurate immune modifier in the tumor to reverse the immune resistance.

## 1 Introduction

Accumulating evidence has suggested that immune suppression in the tumor microenvironment is a major obstacle to effective antitumor therapy in patients.^1^ The suppression of tumor‐specific cytotoxic cells is orchestrated by a variety of immunosuppressive stromal cells, accounting for evading immune surveillance.^1^, ^2^ Therefore, a comprehensive understanding of how the immunosuppressive tumor microenvironment develop is critical and regarded as a promising direction of intervention. Tumor‐associated macrophages (TAMs) constitute the dominant cell population of the tumor microenvironment, derived from the circulating monocytes.^3^ This versatile cell type displays remarkable plasticity with opposite effects on cell survival, immune responses, and angiogenesis, causing overall pro‐ or antitumor outcomes.^4^ Macrophages are known for their vital role in immune defense against the foreign pathogens to prevent tumorigenesis.^5^ However, an increasing number of studies indicated that TAMs skew from proinflammatory M1‐like phenotype in early stages of some tumors toward the anti‐inflammatory M2‐like phenotype in most advanced tumors, promoting the tumor growth, angiogenesis, and metastasis.^4^, ^6^ Moreover, the increased infiltration of M2‐like TAMs also provides a protumor immunosuppressive milieu by altering recruitment and function of leukocytes, such that it is associated with therapeutic failure and poor clinical prognosis in almost all tumors.^4^, ^5^, ^6^ Nevertheless, the mechanisms underlying the modulation of TAMs' phenotype in tumor progression, especially after antitumor therapy is yet to be elucidated.

Hypoxia is a vital environmental cue that induces macrophage trafficking into tumor areas.^7^ Furthermore, the level of specific M2‐like phenotype macrophage is significantly increased in response to hypoxia, which promotes cancer malignancy and progression.^8^ Additionally, TAMs in the hypoxic milieu mediate the resistance of antitumor drugs and tumor relapse.^9^ Hence, hypoxia is a potent stimulus of the tumor microenvironment as it limits the ability of the immune system and subverts the immune responses to induce pro‐tumor immunosuppressive effect.[qv: 7a,9]

Vascular disrupting agents (VDAs) constitute the potential class of antivascular therapy. Recently, these VDAs have gained increasing attention due to their entry into clinical trials and showing therapeutic efficacy.^10^ The VDAs, selectively targeting the established tumor vasculature, cause a rapid and severe shutdown of blood vessels, ultimately resulting in secondary tumor cell necrosis.^10^, ^11^ Combretastatin A4 (CA4), the leading VDA, is a microtubule‐depolymerizing agent leading to cytoskeletal destabilization, followed by morphological changes in the endothelial cells.^12^ Furthermore, with respect to the rapid clearance of CA4 from tissues and plasma, we utilized the nanocarrier‐based drug delivery systems to develop a nanosized polymeric CA4 prodrug (PLG‐CA4), which showed a high concentration and prolonged retention inside cancer, resulting in effective vascular disruption.^13^ Importantly, this agent was firstly realized distribution around the tumor vessels due to the low tissue permeability in solid cancers, which markedly enhanced therapeutic efficiency as compared to the small molecular prodrug of CA4 (combretastatin‐A4 phosphate). While several studies have demonstrated that the administration of VDAs significantly increases the level of intratumoral hypoxia in solid tumors.^14^ Upon hypoxia‐induced chemoattractant action, the tumor microenvironment is invariably remodeled to limit the therapeutic efficacy of VDAs.[qv: 14a,b,15] However, the specific role of VDAs in the immunosuppressive effect still be unrevealed.

**Scheme 1:**
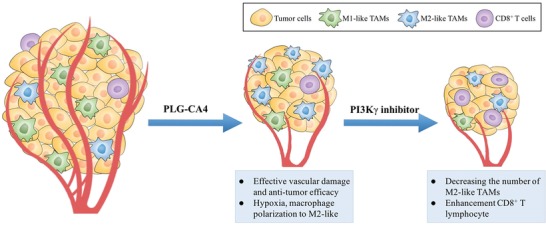
Schematic illustration of combination of PLG‐CA4 and PI3Kγ inhibitor. PLG‐CA4 induces effective vascular damage and antitumor efficacy, while increases the level of hypoxia and reshapes of macrophage to M2‐like. Selective inhibition of PI3Kγ decreases M2‐like macrophages numbers and enhances CD8^+^ T lymphocytes.

Here, we found that PLG‐CA4 induced the polarization of TAMs toward the M2‐like phenotype in 4T1 mammary tumors, which restrained the antitumor activity. In order to attenuate these immunosuppressive effects, we used the phosphoinositide 3‐kinase gamma isoform (PI3Kγ) selective inhibitor in synergy with PLG‐CA4, which significantly decreased the number of M2‐like TAMs and potentially enhanced of the cytotoxic T lymphocytes (CTLs). Also, the combination of PLG‐CA4 and PI3Kγ inhibitor markedly improved the tumor therapeutic effect of NLG919, an inhibitor of immune checkpoint indoleamine 2,3‐dioxygenase (IDO). In the current study, PI3Kγ inhibitor attenuates the immunosuppressive effect of PLG‐CA4, which significantly prevented the tumor development and prolonged the survival (**Scheme** **1**). Our findings provide preclinical evidence that support the rationale for targeting PI3Kγ inhibitor with PLG‐CA4 for the treatment of metastatic breast cancer.

## 2 Results

### 2.1 PLG‐CA4 Induces Effective Vascular Damage, While Increases the M2‐Like Macrophage Numbers

**Figure 1:**
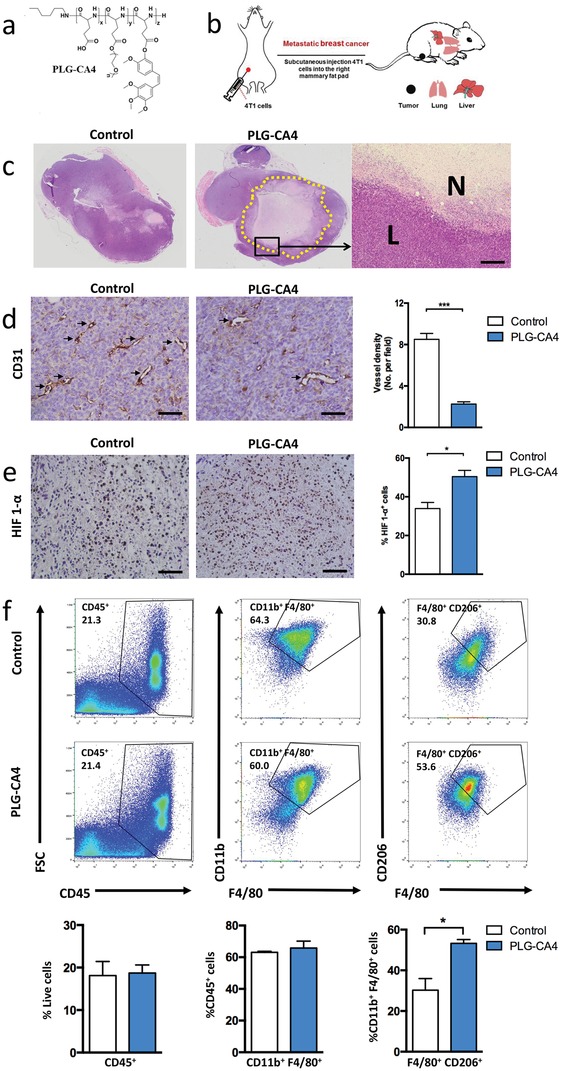
PLG‐CA4 induces macrophages polarization to the M2 phenotype. a) Chemistry structural formula of PLG‐CA4. b) Schematic illustration of metastatic 4T1 mammary carcinoma models. c) H&E analysis of whole tumor tissues at 48h after a single intravenous injection with PLG‐CA4 at a CA4 dose of 35.0 mg kg^−1^; magnification of area outlined at the right (scale bar, 200 µm), L and N indicate the live and necrotic region. d) Representative images of CD31 staining (black arrow) of tumor sections at 48 h after PLG‐CA4 injection (scale bar, 50 µm), the microvessel density (MVD) quantified by counting positive signals in five randomly selected fields (400×) using Image J Software. e) Representative images of HIF 1‐α staining of tumor sections at 48 h after PLG‐CA4 injection (scale bar, 50 µm), HIF 1‐α^+^ cells percentage by counting positive cells in five randomly selected fields (400×) using Image J Software. f) Representative flow cytometric analysis and quantification of CD45^+^ (TIL), CD11b^+^F4/80^+^ (TAM), and F4/80^+^CD206^+^ (M2) cell populations in 4T1 tumors at 48 h after PLG‐CA4 injection. The histogram bars show the percentage of each cell population (*n* = 3). Data were presented as means ± s.e.m. *p < 0.05, **p < 0.01, ***p < 0.001.

In order to investigate the role of PLG‐CA4 (**Figure** 1a) in both orthotopic cancer and metastasis, we selected the well‐characterized 4T1 murine mammary carcinoma model (Figure 1b). The PLG‐CA4 nanomedicine was synthesized from poly(l‐glutamic acid)‐*graft*‐methoxy poly(ethylene glycol) copolymer (PLG‐*g*‐mPEG) and CA4 by the Yamaguchi esterification reaction. We analyzed the therapeutic effect and vascular‐disrupting efficacy of PLG‐CA4 in 4T1 tumor‐bearing mice. At 48 h after a single injection of PLG‐CA4, tumor necrotic areas including fragmented nuclei and infiltrated inflammatory cells were significantly observed in PLG‐CA4‐treated tumors (Figure 1c). The microvessel density (MVD) was assessed by CD31 staining, which was markedly decreased in tumor tissues with VDA therapy, suggesting the effective vascular damage and antitumor efficacy (Figure 1d). While PLG‐CA4 induced a significantly high level of hypoxia‐inducible factors 1‐α (HIF1‐α) translocated in the nucleus (Figure 1e), which was stabilized only under hypoxic conditions.^9^ The hypoxic tumor microenvironment is known to subvert the function of macrophages through direct or indirect regulation by tumor cells,^16^ and thus, we examined the number of tumor‐infiltrating macrophages as well as the phenotype modulation. TAMs (CD11b^+^F4/80^+^) constituted the major population of CD45^+^ tumor‐infiltrating leukocytes (TILs) in the 4T1 mammary carcinoma model (Figure 1f), which was consistent with evidence reported in several studies.[qv: 3,14a] Although PLG‐CA4 did not affect the total TILs and TAMs cell population in tumors, it enhanced the accumulation of immunosuppressive M2‐like (CD11b^+^F4/80^+^CD206^+^) state (Figure 1f), indicating that PLG‐CA4 treatment induced macrophage polarization to TAM‐M2 phenotype (Control 30% vs PLG‐CA4 53%; *p* < 0.05). To the best of our knowledge, this is the first provided evidence for VDAs treatment inducing the reshaping of macrophages to M2‐like phenotype.

### 2.2 Selective Inhibition of PI3Kγ Decreases M2‐Like Macrophages Numbers in Tumor

**Figure 2:**
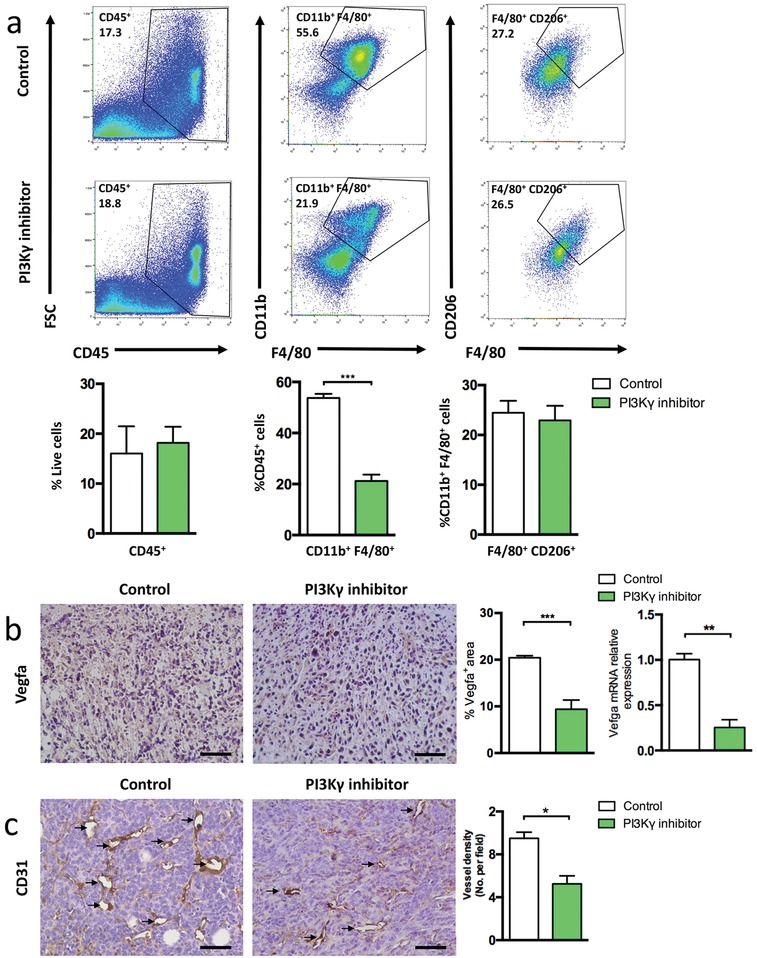
PI3Kγ inhibitor decreases TAM‐M2 recruitment in tumors. a) Representative flow cytometric analysis and quantification of CD45^+^ (TIL), CD11b^+^F4/80^+^ (TAM), and F4/80^+^CD206^+^ (M2) cell populations in 4T1 tumors at 7 days after TG100‐115 treatment. The histogram bars show the percentage of each cell population (*n* = 3). b) Representative images of Vegfa staining of tumor sections at 7 days after PI3Kγ inhibitor treatment (scale bar, 50 µm), percentage areas of Vegfa^+^ signals in five randomly selected fields (400×) using Image J Software. mRNA levels of Vegfa in tumor samples (*n* = 3). c) Representative images of CD31 staining of tumor sections at 7 days after PI3Kγ inhibitor treatment (scale bar, 50 µm). The microvessel density (MVD) quantified by counting positive signals in five randomly selected fields (400×) using Image J Software. Data were presented as means ± s.e.m. *p < 0.05, **p < 0.01, ***p < 0.001.

Next, we tested whether PI3Kγ inhibition reduced the number of immunosuppressive M2‐like macrophages in the tumor microenvironment to enhance the therapeutic efficacy of PLG‐CA4. PI3Kγ is a molecular switch that controls immune suppression, thus PI3Kγ inhibition controls the recruitment of macrophages and converts them to antitumor immune responses.^3^, ^17^ Hence, the selective inhibitor of PI3Kγ, TG100‐115, was used in the 4T1 breast cancer‐bearing mouse model.[qv: 17a,18] Compared to the control group, the accumulation of TILs in the tumors did not differ significantly in the PI3Kγ inhibitor‐treated group (**Figure** 2a). The PI3Kγ inhibition reduced the total macrophage trafficking into tumors without altering the M2‐phenotype ratio, thereby suggesting a significant decrease in the number of immunosuppressive TAM‐M2 (Figure 2a). Additionally, the expression of vascular endothelial growth factor A (VEGF‐A) derived from the tumors was significantly reduced after treatment with PI3Kγ inhibitor (Figure 2b). Consistent with the protein expression, the level of *Vegfa* mRNA was also markedly decreased in tumors from PI3Kγ inhibitor‐treated mice (Figure 2b). Several studies demonstrated the critical role of VEGF‐A in tumor angiogenesis, which is considered as the primary stimulus.^19^ The level of MVD was also significantly lower in the PI3Kγ inhibition group than that in the control group, indicating that tumor angiogenesis was associated with immunosuppression (Figure 2c).

### 2.3 PLG‐CA4 Synergizing with PI3Kγ Inhibitor Suppresses Tumor Growth

**Figure 3:**
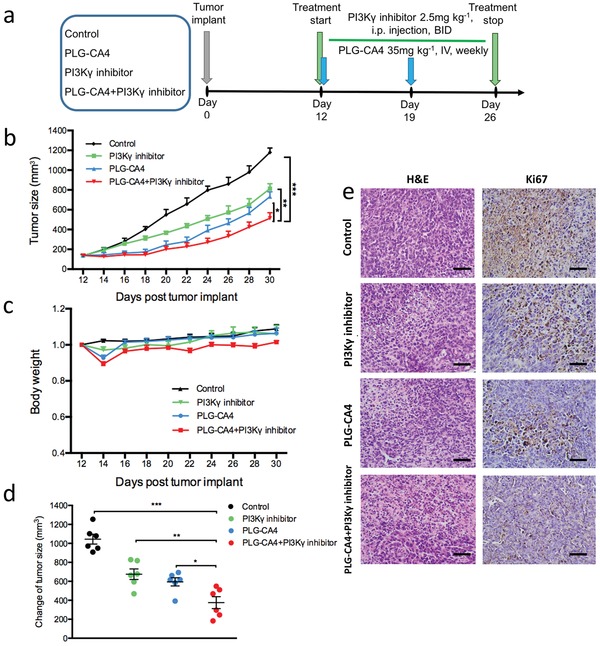
The combination of PLG‐CA4 and PI3Kγ inhibitor suppresses the growth of orthotopic breast cancer. a) Therapy regimen. b,c) Mean tumor size and body weight of subcutaneous 4T1 tumor in Control, PI3Kγ inhibitor, PLG‐CA4 or PI3Kγ inhibitor combination with PLG‐CA4 (*n* = 6). d) Change of tumor sizes on day 30 of regime. e) H&E and Ki67 staining of tumor sections on day 30 of therapy regimen (scale bar, 50 µm). Data were presented as means ± s.e.m. *p < 0.05, **p < 0.01, ***p < 0.001.

To evaluate the effect of combination treatment with PLG‐CA4 and PI3Kγ inhibitor, we conducted the antitumor therapy regimen in 4T1 orthotopic mammary tumor metastasis model (**Figure** 3a). Both PLG‐CA4 and PI3Kγ inhibitor significantly delayed the tumor growth as compared to the control group alone (Figure 3b). PLG‐CA4 was injected once a week, which markedly inhibited the tumor growth, indicating a rapid and durable effect of vascular damage inside the tumors. Importantly, the enhanced tumor inhibition was observed in the combination treatment group (Figure 3b), suggesting that PI3Kγ inhibitor improved the therapeutic efficacy of PLG‐CA4. On day 30 of experiments, the treatment with PLG‐CA4 plus PI3Kγ inhibitor led to a considerably reduced tumor suppression rate as compared to each group of study (Figure 3d). Furthermore, no significant loss of body weight was observed in the combined therapy group, indicating low side effects of this therapeutic strategy (Figure 3c). To further assess the morphological changes in the tumor tissues, the tumors were isolated from mice and stained for pathological analysis on day 30. The maximal necrotic area was observed in the combination‐treated mice (Figure 3e). Ki67, a nuclear protein required to maintain the individual mitotic chromosomes dispersed during the cell cycle, presented the most intensive signals in the combined therapy group as assessed by staining (Figure 3e). These results demonstrated that the therapeutic strategy of PLG‐CA4 and PI3Kγ inhibitor combination markedly suppresses the tumor growth.

### 2.4 PLG‐CA4 Synergizing with PI3Kγ Inhibitor Reduces Tumor Metastasis

**Figure 4:**
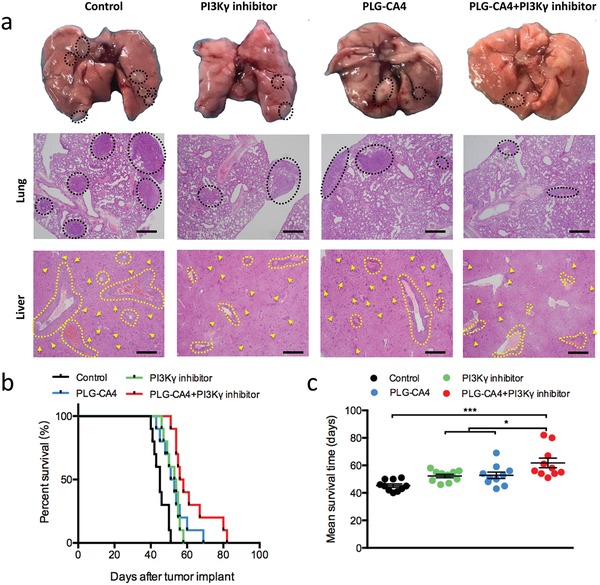
The combination of PLG‐CA4 and PI3Kγ inhibitor reduces the breast cancer metastasis. a) Representative images of lungs and representative H&E sections of lung and liver for the different groups on day 30 of therapy regimen (scale bar, 500 µm). The dashed lines and yellow arrows indicated the metastasis foci. b,c) Overall survival and mean survival time of 4T1 tumor‐bearing mice treated with Control, PI3Kγ inhibitor, PLG‐CA4 or PI3Kγ inhibitor combination with PLG‐CA4 (*n* = 10). Data were presented as means ± s.e.m. *p < 0.05, **p < 0.01, ***p < 0.001.

Immunosuppressive TAM‐M2 induces metastasis‐promoting functions; however, whether vascular targeting agents slow down the metastatic disease progression, as well as the overall survival is yet controversial.^4^, ^16^, ^20^ Therefore, we evaluated the tumor metastasis in highly invasive and aggressive orthotopic breast cancer model 4T1, which spontaneously metastasizes to distant organs. It is considered that fatal pulmonary metastasis might result in sudden death among cancer patients.^21^ The control mice gradually developed dyspnea and died due to a large number of metastatic sites in the lungs on day 30 of therapy regimen (**Figure** 4a). While the therapeutic agent‐treated groups presented a reduction in the pulmonary metastatic sites (Figure 4a). Notably, it can be observed that the combination of PLG‐CA4 and PI3Kγ inhibitor weakened the lung metastasis, represented by the pathological changes in the susceptible organs (Figure 4a). Although monotherapy reduced the breast cancer metastasis to some degree; only minimal signal of metastasis and a relatively intact structure was detected in the mice treated with PLG‐CA4 plus PI3Kγ inhibitor (Figure 4a). Conversely, the multifocal metastasis of the organs and metastatic cells diffusing inside the hepatic vessels was observed in the control mice (Figure 4a).

Since metastasis is tightly associated with tumor burden and the overall survival in breast cancer,[qv: 21b] we further analyzed the effect of the combination of PLG‐CA4 and PI3Kγ inhibitor on the survival time. Compared to the control group, all the drug‐treated mice were benefited with prolonged overall survival and average survival time (Figures 4b,c). Importantly, PI3Kγ inhibition synergizing with PLG‐CA4 significantly extended the mean survival time from 52 days in the mice treated by monotherapy to 61.8 days (Figures 4b,c). Taken together, these results suggested that PLG‐CA4 plus PI3Kγ inhibitor effectively prevented cancer metastasis and prolonged the survival time in addition to inhibiting the primary tumor growth.

### 2.5 Mechanisms of Antitumor Performance by Attenuating Immunosuppressive Effect

**Figure 5:**
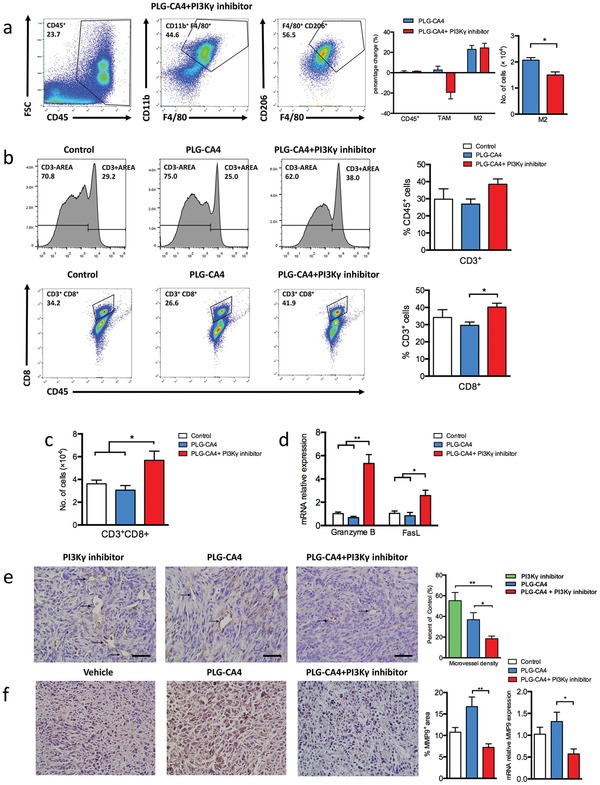
Mechanisms of improved therapeutic efficacy by attenuating the immunosuppressive effect. a) Representative flow cytometric analysis and quantification of CD45^+^ (TIL), CD11b^+^F4/80^+^ (TAM), and F4/80^+^CD206^+^ (M2) cell populations in 4T1 tumors treated PLG‐CA4 combined PI3Kγ inhibitor at day 14 of therapy regimen. The left histogram bars show the percentage change of each cell population (CD45^+^, TAM, M2) in therapy group compared to control group (*n* = 3). The right histogram bars show the count of M2 (*n* = 3). b) Representative flow cytometric analysis and quantification of CD3^+^, CD3^+^CD8^+^ (CTL) cell populations treated with Control, PLG‐CA4 or PI3Kγ inhibitor combined PLG‐CA4 at day 14 of therapy regimen. c) The count of CD3^+^CD8^+^ (CTL) at day 14 of therapy regimen (*n* = 3). d) mRNA levels of Granzyme B and FasL in tumor samples (*n* = 3). e) Representative images of CD31 staining of tumor sections at day 14 of therapy regimen (scale bar, 50 µm). The microvessel density (MVD) quantified by counting positive signals in five randomly selected fields (400×) using Image J Software. f) Representative images of MMP9 staining of tumor sections at day 14 of therapy regimen (scale bar, 50 µm), percentage areas of MMP9^+^ signals in five randomly selected fields (400×) using Image J Software. mRNA levels of MMP9 in tumor samples (*n* = 3). Data were presented as means ± s.e.m. *p < 0.05, **p < 0.01, ***p < 0.001.

Compared to the monotherapy of PLG‐CA4, the combination of PLG‐CA4 and PI3Kγ inhibitor markedly suppressed the recruitment of circulating macrophages to tumors, thereby significantly lowering the level of immunosuppressive TAM‐M2 (2.0 × 10^4^ to 1.5 × 10^4^ per tumor) (**Figure** 5a). Several studies demonstrated that PI3Kγ blockade in macrophages stimulates the pro‐inflammatory responses to promote the adaptive immunity.^17^ Compared to the monotherapy, the number of infiltrating CD8^+^ T lymphocytes was markedly increased in mice treated with PLG‐CA4 plus PI3Kγ inhibitor (3.0 × 10^4^ to 5.7 × 10^4^ per tumor), suggesting that less immunosuppressive phenotype of the macrophages was tightly associated with CTLs trafficking into the tumors (Figures 5b,c). Additionally, the high expression of granzyme B and FasL in cytotoxic cells of the tumor microenvironment was obviously observed in the combination‐treated mice (Figure 5d). Although the decrease in the number of M2‐like macrophages was only one quarter, both the number of CD8^+^ cells and the function of cytotoxic cells was significantly increased after the combined therapy (Figures 5c,d). The critical role of tumor‐infiltrating macrophages in promoting tumor angiogenesis is strongly established.^22^ Compared to PLG‐CA4 alone, the CD31‐positive signals were further reduced, and the inter‐tumor microvessels were poorly structured after treatment with PI3Kγ inhibitor (Figure 5e). Tumor aggression and metastasis were increasingly characterized as the dynamic correlation between the tumor cells and tumor microenvironment, while matrix metalloproteinases (MMPs) serve as potential biomarkers of tumor progression and metastatic spread in this process, for example, MMP9.^23^ Furthermore, the expression of MMP9 protein and gene was significantly decreased in tumor tissues treated with PLG‐CA4 and PI3Kγ inhibitor combination (Figure 5f).

### 2.6 Combination of PLG‐CA4 and PI3Kγ Inhibitor Enhances the Therapeutic Effect of IDO Inhibitor

**Figure 6:**
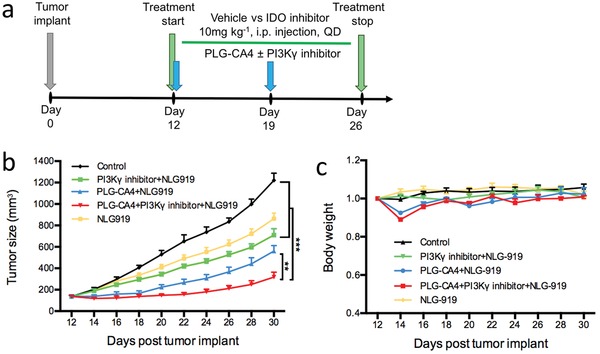
The combination of PLG‐CA4 and PI3Kγ inhibitor enhances antitumor therapeutic efficacy of NLG919. a) Therapy regimen. b,c) Mean tumor size and body weight of subcutaneous 4T1 tumor in Control, IDO inhibitor, PI3Kγ inhibitor and/or PLG‐CA4 combined with IDO inhibitor (*n* = 6). Data were presented as means ± s.e.m. *p < 0.05, **p < 0.01, ***p < 0.001.

To determine whether the combination of PLG‐CA4 and PI3Kγ inhibitor interacts with other immune therapies, we used the checkpoint inhibitor of IDO (NLG919) in 4T1 breast tumor models (**Figure** 6a).^24^ IDO is a crucial negative feedback protein in generating immunosuppressive molecules that inhibit the effector T cells and improve the regulatory T cells.[qv: 24c,25] Single agent of IDO inhibitor seemed to be an ineffective therapy for controlling the tumor progression with a decrease in the tumor burden to a lesser extent (Figure 6b). Compared to the treatment with IDO inhibitor alone, the combination of PLG‐CA4 and PI3Kγ inhibitor significantly suppressed the growth of breast cancer, as well as the durable tumor suppression rate (Figure 6b). In addition to the improved therapeutic effect, the combination of PLG‐CA4 and PI3Kγ inhibitor to IDO inhibitor did not cause an obvious loss of body weight, suggesting fewer side effects of this treatment (Figure 6c). Thus, these results demonstrated that the combination of PLG‐CA4 and PI3Kγ inhibitor markedly improves the antitumor response of IDO antagonist, which significantly induces tumor regression.

## 3 Discussion

Breast cancer is the most common malignancy and the second leading cause of cancer‐related mortality in women worldwide.^26^ The incidence of synchronous distant metastasis in newly diagnosed breast cancer is up to 10%,^27^ and the five‐year survival rates for the diagnosed patients dramatically reduces from 99% to 26% due to distant metastasis.^28^ Metastasis is a multistep process, wherein solid cells spread from the original site to distant organs through tumor vasculature and deemed as the collaborative interaction between malignant cells and immunosuppressive stromal cells.^29^ The tumor vasculature and stromal cells are the two key components of the tumor microenvironment, and targeting these might be a beneficial therapeutic strategy to inhibit the tumor growth and metastasis.

During tumor progression, the “angiogenic switch” is activated, which in turn, continually sprouts new vessels, supplying oxygen and nutrients for the neoplastic cells.^30^ VDAs disrupt the existing tumor vasculature to shut down the blood perfusion and resulting in tumor cell necrosis.^10^ CA4 is the leading VDA and has effective antitumor ability via interfering the dynamics of tubulin, while CA4 has a poor water‐soluble and short circulation half‐life.^31^ So far, nanocarriers has the favorable physicochemical properties via enhanced permeability and retention (EPR) effect, leading to significant improvement of chemical drugs.^32^ The CA4 loaded Ala‐Pro‐Arg‐Pro‐Gly‐poly(ethylene glycol)‐poly(d,l‐lactide)/monomethoxy poly(ethylene glycol)‐poly(d,l‐lactide) (APRPG‐PEG‐PDLLA/MPEG‐PDLLA) mixed micelles have demonstrated a significant inhibition in breast cancer.^31^ Additionally, a novel peptide specifically binding to VEGF receptor was also found to suppress angiogenesis in vitro and in vivo.^33^ Importantly, we developed poly(l‐glutamic acid)‐combretastatin A4 conjugate inducing a rapid and prolonged effect of vascular disruption inside the tumors, markedly reducing the tumor burden.

However, clinical trials have shown that the addition of vascular‐targeted drugs cannot achieve the expectant therapeutic benefit in progression‐free survival or overall survival in women with advanced breast cancer.^34^ Thus, combining the results of the published studies with those of the current study revealed that hypoxia‐regulated and immune‐mediated programs are accomplices in resistance to vascular‐targeted therapy, especially the immunosuppressive TAMs.[qv: 14a,35] Therefore, converting the immunosuppressive macrophages to antitumor effect has been regarded as the novel therapeutic opportunity to improve VDAs, as well as the antiangiogenic therapy.

Currently, immunotherapy has focused on the blockade of immune checkpoint signals on cytotoxic cells; however, most of the clinical trials on checkpoint blockages in solid tumors could not yield positive outcomes.^16^, ^36^ This phenomenon could be explained as follows. First, emerging evidence indicated that the imbalance between the tumor burden and T‐cell invigoration might be responsible for the clinical failure, implying that even robust reinvigoration induced by immunotherapy could be clinically ineffective in case of high tumor burden.^36^ Second, both tumor‐promoting inflammatory cells and vascular endothelial cells constitute the major cell population of the tumor microenvironment, and the interaction of these cells play a pivotal role in tumor immune response.^16^ Thus, vascular‐targeted therapy enhances the clinical benefit with immune response modifier and vice versa.^16^, ^35^ In this study, we focused on PLG‐CA4‐disrupted tumor vessels to limit the tumor growth and TG100‐115‐regulated macrophages to reshape the tumor microenvironment.

We showed that VDA‐treatment‐induced hypoxia triggers the protumor effects in macrophages that might stimulate the production of angiogenic cytokines and matrix‐regulatory factors. Interestingly, a previous study has implicated that macrophage‐dominant PI3Kγ controls the stability of hypoxia‐induced HIF1‐α and HIF2‐α, and directs the tumor proliferation, angiogenesis, and metastasis.^37^ Genetic or pharmacological inhibition of p110γ in mice models significantly decreases the number of HIFα and its related downstream transcription targets in response to hypoxia. In addition, hypoxia‐derived exosomes mediated the differentiation and polarization of macrophages via the activation of the PI3Kγ signaling pathway, which then leads to the invasion, migration, and epithelial–mesenchymal transition in pancreatic cancer, as well as distant organ metastasis.^38^ This phenomenon might explain the ability of PI3Kγ inhibitor to reverse the hypoxia‐induced immunosuppression by modifying the state of macrophages in tumors.

PI3Kγ belongs to the class I PI3K lipid kinase family, which response extracellular stimuli into intracellular signals to regulate several critical biological functions including cell growth, metabolism, and motility.^39^ In addition to frequent somatic mutations in this pathway, several studies revealed the crucial role of PI3K isoforms in the tumor microenvironment, thereby representing it as a promising therapeutic target.^40^ PI3Kγ is highly expresses in myeloid cells but not cancer cells, and promotes migration of myeloid cell during cancer.^3^, ^17^ Targeting PI3Kγ with a selective inhibitor can reeducate the tumor microenvironment and promote tumor regression without targeting tumor cells directly.[qv: 17b] Thus, we selected a small molecule PI3Kγ antagonist, TG100‐115,[qv: 3,17b] to enhance the therapeutic effect of PLG‐CA4 in 4T1 breast cancer‐bearing mice with a high level of immunosuppressive macrophage infiltration in the tumors. In the current work, we did observe the beneficial effect of PLG‐CA4 and TG100‐115 combination on the inhibition of tumor growth and metastasis without obvious side effects.

Our findings also demonstrated the increased infiltration of CD8^+^ T lymphocytes and the function of cytotoxic cells in the group with combination therapy, indicating that the PI3Kγ signaling pathway in TAMs suppressed the migration of CD8^+^ T cells and the function of cytotoxic cells in the tumors. Several studies further confirmed that inhibition of PI3Kγ blocked the tumor growth via recruiting and/or activation of CD8^+^ T cells, as the PI3Kγ inhibition does not induce an antitumor effect in CD8 null or antibody‐depleted mouse models.^17^ Given the critical role of myeloid cells in antigen presentation, PI3Kγ inhibition did not affect the proportion of effector memory T cells but rather promoted the tumor‐antigen‐specific T cell activation.[qv: 17b] Additionally, in vitro assays showed that neither deletion nor inhibition of PI3Kγ affect the T cell activation and proliferation, suggesting an indirect PI3Kγ‐mediated T cell activation.[qv: 17a] Taken together, PI3Kγ inhibition might recover the CD8^+^ T cell‐mediated cytotoxicity by reshaping the immune suppression to immune stimulation.

Interestingly, we found that the combination of PLG‐CA4 and PI3Kγ inhibitor significantly improved the therapeutic effect of the checkpoint inhibitor NLG919, which is a highly selective IDO antagonist. The overexpression of IDO in tumor cells converts the tryptophan into its metabolites, leading to the death of cytotoxic T cells, while stimulating the function of regulatory T cells.[qv: 24a,25] NLG919 is a small molecule inhibitor of IDO that is currently being evaluated in the clinical trials to increase the T cell response of patients with recurrent advanced solid tumors.^41^ The current results suggested that PLG‐CA4 targeting the tumor vasculature significantly decreases the tumor burden, and PI3Kγ inhibitor regulating the macrophages further reverses the immunosuppressive tumor microenvironment. In addition, the number of infiltrating CD8^+^ T lymphocytes was markedly increased in mice treated with PLG‐CA4 plus PI3Kγ inhibitor. Consequently, the checkpoint inhibitor of IDO might further promote the survival and activity of CD8^+^ T lymphocytes and suppress regulatory T cells, inducing antitumor immunity. There is the synergistic effect between NLG919 and the combination of PLG‐CA4 and PI3Kγ inhibitor, resulting in improved antitumor efficacy. Thus, this finding presented a synergistic effect of the combination of PLG‐CA4 and PI3Kγ inhibitor with other immune therapy.

## 4 Conclusion

In this study, we first found VDAs induced the reshaping of macrophages to M2‐like phenotype in 4T1 metastatic breast cancer. Using PI3Kγ selective inhibitor attenuated the immunosuppressive effect of PLG‐CA4 treatment by decreasing the number of M2‐like TAMs and potential enhancement of CTLs, which significantly prevents tumor development and prolongs the survival. Additionally, the combination of PLG‐CA4 and PI3Kγ inhibitor markedly improved the tumor therapeutic effect of the checkpoint inhibitor NLG919, which is a highly selective IDO antagonist. The current results supported the potential therapeutic strategy of PLG‐CA4 in synergy with the PI3Kγ inhibitor.

## 5 Experimental Section

*PLG‐CA4*: In brief, poly(l‐glutamic acid)‐*graft*‐methoxy poly(ethylene glycol) copolymer (PLG‐*g*‐mPEG) was prepared by an esterification reaction of poly(l‐glutamic acid) (*M_n_* = 20.7 × 10^3^ g mol^−1^, polydispersity index (PDI) = 1.36, polyethylene glycol as the standard) with mPEG5K (*M*_W_ = 5000 g mol^−1^, Aldrich) in a mass ratio of 1:2.^42^ The *M_n_* and polydispersity (polyethylene glycol as the standard) of the obtained PLG‐*g*‐mPEG were 37.3 × 10^3^ g mol^−1^ and 1.91, respectively. The PLG‐CA4 was prepared by the Yamaguchi reaction of PLG‐*g*‐mPEG with combretastatin A4 (CA4) in an dimethylformamide solution at room temperature for 2 h as was previously reported.^13^ The CA4 loading content and hydrodynamic diameter were 33.7 wt% and 36.4 nm, respectively.

*Cell Lines and Cell Culture*: 4T1 murine breast cancer cells were obtained from the Cell Bank of the Chinese Academy of Sciences, and routinely maintained and checked in the School of Basic Medical Sciences, Jilin University. The 4T1 Cells were maintained in Dulbecco's Modified Eagle's Media (DMEM, Gibco) supplemented with 10% fetal bovine serum (FBS, heat‐inactivated, Sijiqing Biotechnology, Hangzhou, China), 50 U mL^−1^ streptomycin, and 50 U mL^−1^ penicillin. The cells were incubated at 37 °C in a humid atmosphere containing 5% CO_2_. The cell density was counted before tumor implant using hemocytometer.

*Animals*: Female Balb/C mice at 6–8 weeks of ages were purchased from Vital River Laboratory Animal Technology Co., Ltd. (Beijing, China). The animal experiments were revised and approved by the Animal Care and Use committee of Jilin University, and all animals received care in accordance with national guidelines and requirement for the Care and Use of Laboratory Animals of Jilin University. Mice were euthanized for signs of distress or before the maximum IACUC allowable tumor size of 1500 mm^3^. Then these animal corpses were preformed Harmless Treatment when the experiments finish.

*Tumor Challenge and Treatment Experiments*: The metastatic breast cancer model was prepared by subcutaneously injecting 2 × 10^6^ 4T1 cells into the right mammary fat pad of the mice on day 0 of experiments. Tumor volume approximately reaching 180 mm^3^ on day 12 post tumor implant, the mice were randomly divided into groups and then treated following the therapy regime. PLG‐CA4 dissolved in phosphate buffered saline (pH 7.4) was at a CA4 dose of 35 mg kg^−1^ by intravenous injection on day 12 and 19 of experiments. PI3Kγ inhibitor TG100‐115 (2.5 mg kg^−1^, twice a day, Selleck) and IDO inhibitor NLG919 (10 mg kg^−1^, once a day, MedChemExpress) were administered by i.p. injection. Treatment was started on day 12 and stopped on day 26 of experiments. The tumor volume was measured every second day with a caliper, using the formula tumor volume = *a* × *b* ^2^/2, where *a* and *b* are the major and minor axes of the tumors. Mice were sacrificed at designed time points for analysis.

*Isolation of Single Cells from Tumors and Flow Cytometry*: Mouse tumor samples were isolated, minced into small pieces with scissors and incubated in Hanks Balanced Salt Solution containing 1 mg mL^−1^ DNase (Sigma), 62.5 µg mL^−1^ collagenase (Roche) and Roswell Park Memorial Institute (RPMI) (Gibco). The digestion was placed for 1 h at 37 °C, including a manual shaking step every 15 min. Following digestion, tumor samples were homogenized by repeated pipetting and then passed through a 100 µm nylon filter in complete RPMI (10% FBS). Red blood cells were disposed with red cell lysis buffer (BD Biosciences) to generate single‐cell suspension. All samples were washed one time and resuspended in PBS to proceed staining.

Single‐cell suspensions were preincubated (20 min, 4 °C) with Fc‐blocking reagent (anti‐CD16/CD32, BD Biosciences) to block nonspecific binding, and then followed by staining (30 min, 4 °C) with appropriate dilutions of various fluorescent‐labeled antibodies: anti‐CD3‐FITC (17A2), anti‐CD4‐APC (RM4‐5), anti‐CD8‐PerCP (53–6.7), anti‐CD45‐PerCP (30‐F11), anti‐CD11b‐APC (M1/70), antibodies purchased from BD Biosciences; anti‐F4/80‐FITC (BM8) and anti‐CD206‐PE (MR6F3) antibodies from eBioscience. All data analysis was processed using flow cytometry analysis program FlowJo software (Treestar).

*Hematoxylin and Eosin Staining*: For observing antitumor effect and distant organ metastasis, Balb/C mice bearing 4T1 breast cancer were sacrificed at day 30 of experiments. The tumors and major metastatic organs (liver, lung) were isolated immediately, fixed in 4% PBS buffered paraformaldehyde, and embedded in paraffin. All paraffin‐embedded samples were sectioned at 5 µm thickness and then stained with hematoxylin and eosin (H&E). Histological alterations were taken by microscope (Olympus CKX41).

*Immunohistochemical Staining*: Immunohistochemical staining was performed as the two‐step immunocytochemistry protocol (Zhongshan Goldbridge Biotechnology, Beijing, China). Briefly, tumor samples were fixed in 4% PBS buffered paraformaldehyde, embedded in paraffin, and then sectioned at 5 µm thickness. All sections were carried out to antigen retrieval and block nonspecific binding. Then, the sections were incubated with anti‐CD31 (ab28364), anti‐HIF‐1 alpha (ab2185), anti‐MMP9 (ab38898), anti‐VEGFA (ab52917), anti‐ki67 (ab15580) antibodies purchased from Abcam, followed by incubation with a peroxidase‐labeled goat antirabbit IgG secondary antibody. After moderate washing, samples were stained with DAB and counterstained with hematoxylin. Histological images were observed by microscope (Olympus CKX41) and quantitative analyses were used by ImageJ software (NIH, Bethesda, MD, USA).

*Quantitative RT‐PCR*: Total RNA from tumor tissues was isolated with Total RNA Purification Kit (GeneMark), and 1 µg of total RNA was reverse‐transcribed to single‐stranded cDNA by the First‐Strand cDNA Synthesis SuperMix (TransGen). Sybr green‐based qPCR was performed using murine primers to *Vegfa*, *Mmp9*, *Gzmb, Fasl* (Sangon Biotech). The expression of mRNA levels was normalized to *Gapdh* (dCt = Ct gene of interest − Ct *Gapdh*). Relative mRNA expression was calculated by the method (ddCt = 2^^−(dCt sample – dCt control)^).

*Statistical Analysis*: All data were expressed as means ± s.e.m. Statistical significance was analyzed by using one‐way ANOVA, followed by Tukey or Newman–Keuls post hoc analysis. One‐way ANOVA was performed to test mean differences between two or more groups to compare every mean with every other mean. The analyses were performed with GraphPad Prism 5.0 statistical software (GraphPad software, San Diego, Calif). *p < 0.05 was considered to indicate statistical significance, **p < 0.01 was highly significant difference and ***p < 0.001 extremely significant difference. All experiments were performed at least twice times, n refers to biological replicate.

## Conflict of Interest

The authors declare no conflict of interest.
